# Macrophage-targeted PEGylated liposomes ameliorate experimental autoimmune encephalomyelitis

**DOI:** 10.3389/fimmu.2025.1657131

**Published:** 2026-01-15

**Authors:** Alexander Muselman, Lewis W. Yu, Khoa D. Nguyen, Mohammed Inayathullah, Qiang Liu, Kyle D. Brewer, Andrey V. Malkovskiy, Jayakumar Rajadas, Edgar G. Engleman

**Affiliations:** 1 Department of Pathology, Stanford University, Stanford, CA, United States; 2 Advanced Drug Delivery and Regenerative Biomaterials Laboratory of Cardiovascular Institute, Stanford University School of Medicine, , Stanford, CA, United States; 3 Department of Plant Biology, Carnegie Institute for Science, Stanford, CA, United States; 4 Department of Bioengineering and Therapeutic Sciences, University of California San Francisco, San Francisco, CA, United States; 5 Stanford Cancer Institute, Stanford University, Stanford, CA, United States

**Keywords:** experimental autoimmune encephalomyelitis (EAE), liposome, macrophages, multiple sclerosis, neurodegenerative disease, neuroinflammation

## Abstract

Macrophages are the predominant immune cell type found in active multiple sclerosis (MS) and experimental autoimmune encephalomyelitis (EAE) lesions, where they contribute to demyelination and axonal damage. Depending on the lesion stage, these cells can exhibit either a pro-inflammatory or neurotoxic phenotype that drives central nervous system (CNS) injury or an anti-inflammatory phenotype that promotes remyelination. Therefore, strategies that modulate macrophage function may offer therapeutic benefits for MS. Polyethylene glycol (PEG) has shown anti-inflammatory and neuroprotective effects in various models of inflammation and neurodegeneration, but the mechanisms involved remain poorly understood. In this study, we investigated the potential of PEG and PEG-based delivery systems to modulate EAE. Although PEG alone did not alter EAE progression, it suppressed the pro-inflammatory phenotype of macrophages *in vitro*. Given the clinical potential and macrophage-targeting properties of larger PEGylated liposomes, we assessed the impact of large (~700 nm) PEGylated liposomes in EAE. These liposomes selectively targeted activated, CNS-infiltrating macrophages and, when administered to mice either before or after neurological manifestations of EAE had developed, they significantly reduced both clinical signs as well as demyelination in the spinal cord. Mechanistically, this treatment reduced macrophage secretion of pro-inflammatory cytokine IL-1β and decreased macrophage and T cell infiltration into the CNS compared to untreated controls. Together, these findings highlight the therapeutic potential of macrophage-targeted PEGylated liposomes in controlling IL-1β-mediated neuroinflammation in MS and potentially other neurodegenerative diseases.

## 1 Introduction

Multiple sclerosis (MS) is a chronic, immune-mediated disorder of the central nervous system (CNS) characterized by inflammation, demyelination, and subsequent neurodegeneration, leading to diverse neurological disease manifestations depending on lesion location and severity (1–3). A variety of immune cells—including T cells, B cells, neutrophils, and macrophages—contribute to MS pathogenesis (4–7). Among these, macrophages—including CNS-resident microglia, non-parenchymal border-associated macrophages, and infiltrating monocyte-derived macrophages—are the predominant immune cell type found in all MS lesions (7–9). In active MS lesions, pro-inflammatory macrophages promote demyelination by stimulating and activating other immune cells, such as T cells, and by releasing neurotoxic mediators (10–12). In contrast, within remyelinating lesions, anti-inflammatory macrophages facilitate tissue repair by dampening inflammation, clearing myelin debris, and secreting neurotrophic factors that support oligodendrocyte maturation and remyelination (10–12).

The plasticity and versatility of macrophages position these cells as promising therapeutic targets for MS, given their potential to inhibit their inflammatory activity and even reprogram them into anti-inflammatory cells that dampen inflammation and demyelination (13–15). Although no approved MS therapies directly target macrophages or microglia, preclinical studies in the experimental autoimmune encephalomyelitis (EAE) model show that adoptive transfer of immunomodulatory macrophages or *in vivo* reprogramming of macrophages can suppress inflammation and mitigate neurological disease (16–23). Local macrophage-driven immune responses can sustain adaptive immunity and amplify glial activation within the CNS, potentially contributing to the limited efficacy of current MS treatments that primarily target T and B cells (24). Macrophage-targeted therapies therefore offer a promising strategy to more effectively prevent long-term neurodegeneration.

Polyethylene glycol (PEG) is a versatile polymer of ethylene oxide that has been widely used in pharmaceutical formulations and drug delivery systems due to its ability to reduce the immunogenicity and improve the bioavailability of nanoparticles, drugs, and proteins (25, 26). Beyond its role as a drug carrier, PEG has demonstrated anti-inflammatory and tissue-protective effects in several disease models, including acute necrotizing pancreatitis (27), lethal gut-derived sepsis (28), experimental colitis (29), and spinal cord injury (30). In a prior study, PEG administration prevented EAE development when given prophylactically (31). However, whether PEG is effective as a therapeutic intervention after disease onset and its specific mechanisms of action remain unclear.

Given the anti-inflammatory effects of PEG, the objective of this study was to evaluate the potential of PEG to directly modulate the pro-inflammatory response of macrophages in the EAE model. While PEG itself did not provide therapeutic benefit when administered after disease onset, it reduced pro-inflammatory cytokine production by macrophages *in vitro*. To enhance macrophage-specific targeting *in vivo*, we utilized large (~700 nm) PEGylated liposomes, which preferentially accumulated in macrophages. Treatment of mice with such liposomes suppressed macrophage infiltration of the CNS and reduced the secretion of the pro-inflammatory cytokine IL-1β by macrophages, resulting in significantly reduced EAE severity, whether the liposomes were administered before or after disease onset. These findings underscore the potential clinical utility of macrophage-targeted PEGylated liposomes in neuroinflammatory disease.

## 2 Materials and methods

### 2.1 Mice

Female 9–10 week old C57BL/6J mice (JAX #000664) and SJL/J mice (JAX #000686) were purchased from the Jackson Laboratory. Mice were housed in an animal facility at Stanford University on a 12-hour light/dark cycle. All procedures were approved by the Stanford University Institutional Animal Care and Use Committee and performed in accordance with approved university protocols.

### 2.2 Experimental autoimmune encephalomyelitis induction

EAE was induced in 11–12 week old female mice using Hooke kits. For C57BL/6J mice, the MOG_35-55_/CFA Emulsion PTX kit (EK-2110) was used. Mice were injected subcutaneously with 100 μL of MOG_35-55_/CFA Emulsion in two sites in the back, followed by intraperitoneal injections of 100 ng of pertussis toxin (PTX) four and 24 hours later. For SJL/J mice, the [Ser^140^]-PLP_139-151_/CFA Emulsion PTX kit (EK-2120) was used. Mice were injected subcutaneously with 50 μL of [Ser^140^]-PLP_139-151_/CFA Emulsion in four sites in the back, followed by the same PTX regimen.

### 2.3 Liposome formulation

Liposomes were produced by the ADDReB laboratory of the Stanford Cardiovascular Institute. Briefly, 333 mg of distearoylphosphatidylcholine (DSPC), 67 mg of DSPE-PEG-NH2-3400 (Laysan Bio), and 167 mg of Cholesterol dissolved in chloroform were evaporated and rehydrated with 10 mL of water using a 20-minute path sonicator and probe sonicator for 5 minutes. For the nonPEGylated liposomes, DSPE (18:0/18:0 PE, Echelon Biosciences) was used instead of DSPE-PEG-NH2-3400. To evaluate the giant nanoparticle formation, we used dynamic light scattering (DLS), available in ADDReB (90 Plus Particle Size Analyzer, Brookhaven Instruments Corporation).

### 2.4 Scanning electron microscopy imaging

The samples for SEM were made by drop-casting 10 μL of liposomal solutions on cleaned Si wafers for 10 minutes. The wafers were then gently washed with deionized water twice. The samples were sputtered in a Denton Desk IV sputterer with 10 nm of gold in Argon plasma and imaged in a Quanta 200 SEM (FEI-ThermoFisher) with Everhart-Thornley secondary electrons detector at 30 kV accelerating voltage and 3000X magnification (**Supplementary Figure S1**).

### 2.5 EAE treatments

To assess the ability of PEG to treat EAE, we treated mice daily with 100 μL of 30% PEG 1500 (w/v) in 75 mM Hepes (pH 8.0) solution (Roche) intraperitoneally starting either day 3 or 13 post-EAE induction. To assess the ability of PEGylated liposomes to treat EAE, we treated mice daily with 150 μL of PEGylated liposomes of varying sizes intraperitoneally starting either day 3 or 16 post-EAE induction. Both treatments were continued until the end of the experiment.

To assess the ability of PEGylated liposomes to prevent relapse in SJL/J mice, we treated mice daily with 150 μL of PEGylated liposomes intraperitoneally starting day 13 post-EAE induction. Treatment was continued until day 25 post-EAE induction.

### 2.6 EAE scoring

Classical EAE disease manifestations were scored as follows: 0, normal; 0.5, limp tip of tail; 1.0, limp tail; 1.5, hindlimb inhibition; 2.0, weakness of hindlimbs; 2.5, dragging of hindlimbs; 3.0, complete paralysis of hindlimbs; 3.5, complete hindlimb paralysis and inability to right itself; 4.0, complete hindlimb paralysis and partial forelimb paralysis; 4.5, complete hindlimb and partial forelimb paralysis and no movement around cage; 5.0, moribund. After euthanasia, mice were given a score of 5.0 for the remainder of the experiment. For the spontaneous relapsing-remitting EAE model, relapse was defined as a sustained increase of at least 0.5 in clinical score from the lowest score during remission.

### 2.7 Generation of bone marrow-derived macrophages

Mice were euthanized with CO_2_, and femurs were isolated and crushed in complete Dulbecco’s minimum essential medium supplemented with 10 mM L-glutamine, 100 U/mL penicillin and streptomycin, and 10% fetal bovine serum (cDMEM) using a mortar and pestle. The single-cell suspension was passed through a 70 μm filter, resuspended in ACK Lysing Buffer (Gibco) for 5 minutes, and centrifuged. Cells were then resuspended at a concentration of 2 x 10^6^ cells/mL in cDMEM with 50 ng/mL GM-CSF (PeproTech) for 7 days (37°C and 5% CO_2_). Fresh media with 50 ng/mL GM-CSF was added 2 and 5 days later. By day 7, the bone marrow cells were fully differentiated and used for downstream applications.

### 2.8 Cytokine bead array

BMDMs were plated (1 x 10^5^ cells/well) in a 96-well plate, pre-treated with varying levels of PEG or L-PEGylated liposomes for 2 hours, and stimulated with 500 ng/mL LPS (Invitrogen) overnight (37°C and 5% CO_2_). After 18 hours, supernatant was collected and incubated with anti-IL-1β (BD Biosciences), anti-IL-6 (BD Biosciences) and anti-TNF-α (BD Biosciences) capture and detection antibodies. Samples were then analyzed using a Fortessa Flow Cytometer. Data were analyzed using FlowJo software.

### 2.9 Histology and immunofluorescence

Mice were euthanized with CO_2_ and perfused with 20 mL PBS and 10 mL 4% paraformaldehyde (PFA, Electron Microscopy Sciences) each. Spinal cords were isolated, fixed in 4% PFA for 24 hours, cryopreserved in 30% sucrose (Sigma-Aldrich) for 4–5 days, and frozen in OCT (Fisher Scientific). 14-micron thick sections were processed and stored at -20°C until the day of staining. For immunofluorescence, sections were permeabilized in 0.3% Triton-X100 (Sigma-Aldrich) for 15 minutes at room temperature (RT), blocked with 1% bovine serum albumin (BSA, Sigma-Aldrich), 5% normal goat serum (ThermoFisher), and 5% normal donkey serum (Abcam) for 1 hour at RT, stained with 1:100 rat anti-mouse CD11b (BioLegend, clone M1/70) and rabbit anti-mouse CD4 (clone RM1013, Abcam) overnight at 4°C, incubated with 1:500 goat anti-rat IgG-Cy5 (Abcam) or 1:500 donkey anti-rabbit IgG-TRITC (Abcam) for 1 hour at RT, and then mounted with Vectashield Plus Antifade Mounting Medium with DAPI (Vector Laboratories). For myelin staining, sections were washed in distilled water, dehydrated in 70% ethanol (Sigma-Aldrich), incubated in 0.5% Sudan Black B (Sigma-Aldrich) for 30 minutes, washed in ethanol (70%, 50%, 25%), rehydrated in distilled water, and mounted with 70% glycerol (Sigma-Aldrich). For H&E staining, we used the H&E Stain Kit (Abcam) and mounted with Limonene Mounting Medium (Abcam). Images were then acquired using a Keyence BZ-X1000 microscope (4X or 20X objective lens).

### 2.10 Toxicity studies

To evaluate potential toxicity, we treated mice daily with 150 μL of L-PEGylated liposomes intraperitoneally for 7 days. Mice were weighed on days 0 and 7. On day 7, the mice were euthanized with CO_2,_ and the kidneys, heart, brain, and liver were weighed. The livers were isolated and processed for sectioning as described earlier. We used the H&E Stain Kit (Abcam) and mounted with Limonene Mounting Medium (Abcam). Images were then acquired using a Keyence BZ-X1000 microscope (4X objective lens).

### 2.11 Characterization of CNS immune cells

Mice were euthanized with CO_2_ and perfused with 20 mL PBS each. The spinal cords were then isolated and homogenized in cDMEM using a pre-chilled Dounce homogenizer. The cell suspension was passed through a 70 μm filter and resuspended in 30% Percoll in cDMEM. After centrifugation, cells were labeled with Live/Dead™ Fixable Blue Dead Cell stain (Invitrogen) and Fc block (BioXcell, clone 2.4G2). The cells were then labeled with monoclonal antibodies against cell-specific markers. For intracellular staining of IL-1β, cells were activated *in vitro* with 500 ng/mL lipopolysaccharide (LPS, Invitrogen) in the presence of 5 μg/mL brefeldin A (BioLegend) for 4 hours, permeabilized in Cytofix/Cytoperm (BD Biosciences), and then stained with an anti-IL-1β monoclonal antibody. Stained cells were fixed with 1% PFA and then analyzed using a Fortessa Flow Cytometer. 123 count eBeads™ Counting Beads (Invitrogen) were used to calculate absolute counts. Data were analyzed using FlowJo software. Antibody information is provided in **Supplementary Table S1**. Gating strategy for flow cytometry can be found in **Supplementary Figure S2**.

### 2.12 Characterization of BMDMs

To assess the effect of treatment on macrophage activation, BMDMs were plated (5 x 10^4^ cells/well), incubated overnight, pre-treated with L-PEGylated liposomes for 4 hours, and then stimulated with 100 ng/mL LPS (Invitrogen) overnight (37°C and 5% CO_2_). After 18 hours, the cells were labeled with Live/Dead™ Fixable Blue Dead Cell stain (Invitrogen) and Fc block (BioXcell, clone 2.4G2). The cells were then labeled with monoclonal antibodies against cell-specific markers. For intracellular staining, the cells were permeabilized in Cytofix/Cytoperm (BD Biosciences) and then stained with anti-iNOS and anti-Arg-1 monoclonal antibodies. Stained cells were fixed with 1% PFA and then analyzed using a Fortessa Flow Cytometer. Antibody information is provided in **Supplementary Table S1**.

To assess the effect of treatment on macrophage size, BMDMs were plated (1 x 10^6^ cells/well), incubated overnight, pre-treated with L-PEGylated liposomes for 4 hours, and then stimulated with 100 ng/mL LPS (Invitrogen) overnight (37°C and 5% CO_2_). After 18 hours, the cells were fixed with 4% PFA for 15 minutes, blocked with 1% BSA, 1% goat serum, and 0.1% Fc block for 1 hour, stained with 1:100 rat-anti-mouse CD11b (BioLegend, clone M1/70) for 1 hour, stained with 1:500 goat anti-rat IgG-Cy5 (Abcam) for 1 hour, and then mounted with Vectashield Plus Antifade Mounting Medium with DAPI (Vector Laboratories). Images were then acquired using a Keyence BZ-X1000 microscope (20X objective lens).

### 2.13 Statistical analyses

All statistical analyses were carried out using GraphPad Prism 10 software. All analyses with 2 groups were tested using unpaired 2-tailed *t* tests. All analyses with 3 or more independent groups were tested with an ordinary one-way ANOVA with Tukey’s correction for multiple comparisons. All analyses with two independent variables were tested with a two-way ANOVA with Šidák multiple comparisons. A *p* value less than 0.05 (*) was considered significant; **p < 0.01, ***p < 0.001, and ****p < 0.0001.

## 3 Results

### 3.1 Systemically administered PEG fails to prevent EAE development or treat established disease

**Figure 1:**
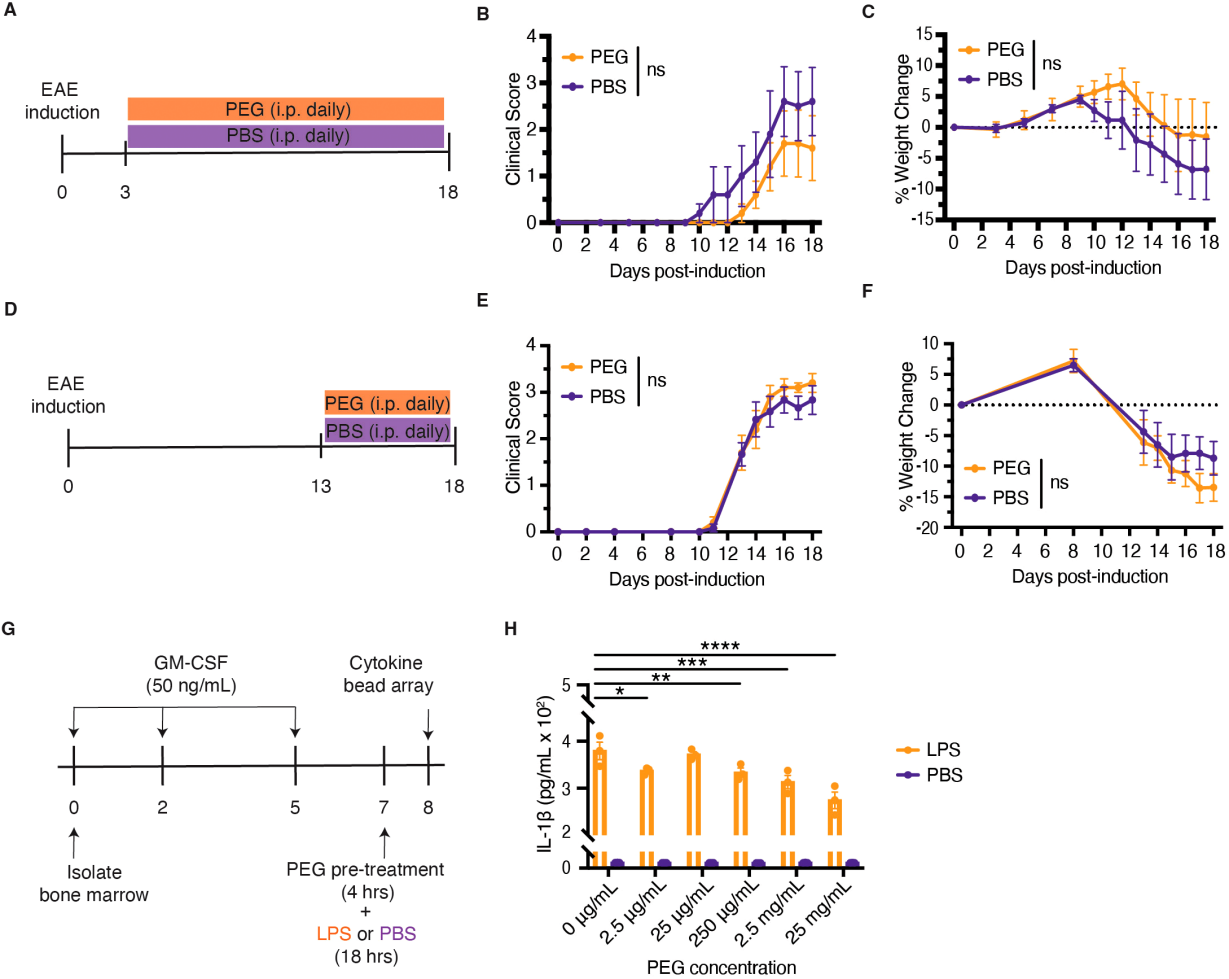
Systemic PEG treatment fails to suppress EAE development and progression, but PEG dampens IL-1β production in macrophages. **(A)** Schematic diagram of daily prophylactic PEG treatment starting day 3 post-initial infusion of MOG_35-55_/CFA. Untreated mice were treated with PBS daily. Mice were monitored for neurological manifestations **(B)** and weight change **(C)** over the course of treatment (n=5 per group). **(D)** Schematic diagram of daily therapeutic PEG treatment starting day 13 post-EAE induction, when mice had an average score of about 1.7. Untreated mice were injected with PBS daily. Mice were monitored for neurological manifestations **(E)** and weight change **(F)** over the course of treatment (n=5–6 per group). **(G)** Timeline of BMDM generation and stimulation for cytokine secretion analysis. After generation, BMDMs were pre-treated with varying concentrations of PEG for 4 hours and then stimulated with LPS for an additional 18 hours. **(H)** IL-1β concentrations in the supernatant were assessed using flow cytometry-based cytokine bead array (n=3 technical replicates per group). Statistical significance was determined using a two-way ANOVA with Šidák multiple comparisons in **(B**, **C**, **E**, **F**, **H)**. Data are represented as mean ± standard error of mean (SEM). Significant changes compared to PEG 0 μg/mL: *p < 0.05, **p < 0.01, ***p < 0.001, and ****p < 0.0001.

We first investigated the effects of systemic PEG prior to EAE development. We induced EAE, treated mice with PEG daily starting day 3 post-induction, and monitored their neurological disease manifestations and weight over time (**Figure 1A**). Mice treated prophylactically with PEG exhibited no change in clinical score (**Figure 1B**) or weight change (**Figure 1C**) compared to untreated mice. Next, we evaluated the effects of systemic PEG on EAE progression after disease manifestations had already developed. We induced EAE, treated mice with PEG daily starting day 13 post-induction, and tracked their neurological disease manifestations and weight over time (**Figure 1D**). Similarly, therapeutic PEG treatment resulted in no change in clinical score (**Figure 1E**) or weight change (**Figure 1F**). These results demonstrate that systemic PEG treatment, whether initiated before or after disease onset, fails to suppress EAE progression.

### 3.2 PEG dampens IL-1β production in bone marrow-derived macrophages

PEG is known to affect many different cell types, including but not limited to enterocytes (32), epithelial cells (29, 33), and neurons (34). PEGylated nanoparticles also suppress the production of pro-inflammatory cytokines and chemokines by macrophages (35). However, these effects were observed under non-inflammatory conditions, and it remains unclear whether they were attributable to PEG itself or to the lipid components of the nanoparticles. Following EAE induction, monocyte-derived macrophages infiltrated the spinal cord and expressed higher levels of pro-inflammatory cytokine interleukin (IL)-1β (**Supplementary Figure S3**). Given the lack of efficacy of PEG in dampening EAE (**Figure 1**), we hypothesized that PEG may not have reached the macrophages in sufficient quantities.

To directly investigate the effects of PEG on macrophages, we pre-treated bone marrow-derived macrophages (BMDMs) with varying concentrations of PEG for four hours and then stimulated them with lipopolysaccharide (LPS). After 18 hours of stimulation, we analyzed the supernatant for pro-inflammatory cytokines using flow cytometry-based cytokine bead array (**Figure 1G**). PEG pre-treatment led to a decrease in IL-1β, but not IL-6, secretion by the BMDMs (**Figure 1H**, **Supplementary Figure S4**). These results demonstrate that PEG can modulate macrophage IL-1β production.

### 3.3 Larger PEGylated liposomes preferentially target monocytes/macrophages and prevent EAE development when administered prior to disease onset

**Figure 2:**
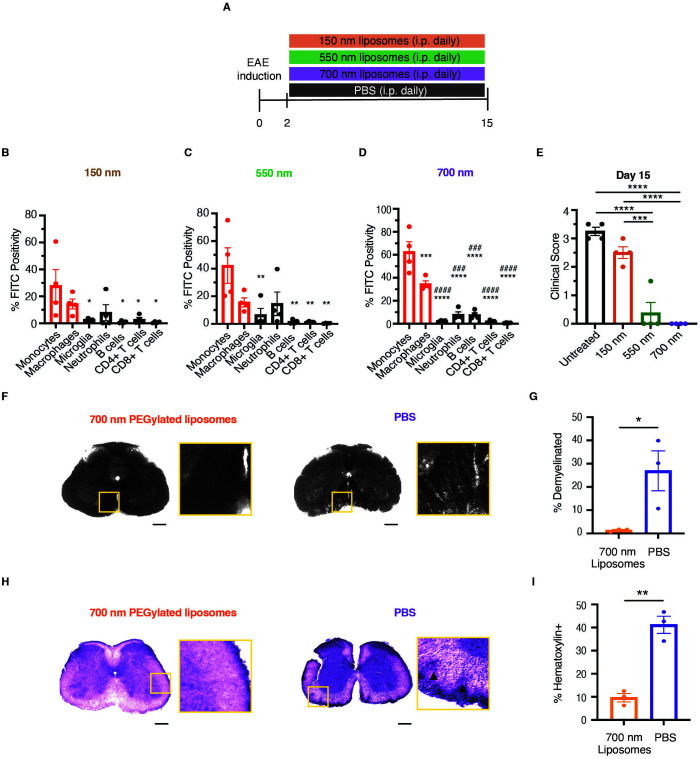
Larger PEGylated liposomes preferentially target monocytes/macrophages and prevent EAE development when administered prior to disease onset. **(A)** Schematic diagram of daily treatment with FITC-labeled PEGylated liposomes of varying sizes (150 nm, 550 nm, or 700 nm) starting day 2 post initial infusion of MOG_35-55_/CFA. **(B–D)** Flow cytometry was then used to determine which immune cells in the spinal cord were targeted by the differently sized liposomes on day 15 post-EAE induction (n=4 per group). **(E)** Clinical scores were determined on day 15 post-EAE induction after daily prophylactic treatments with the differently sized liposomes (n=4 per group). **(F–H)** Spinal cords from 700 nm liposome-treated or untreated mice were isolated on day 15 post-EAE induction. **(F, G)** Demyelination was assessed using Sudan Black B (SBB) staining and **(H, I)** immune cell infiltration was assessed using H&E staining within the white matter on fixed frozen sections (n=3 per group). Triangles denote regions of dense immune cell infiltration. Scale bar, 200 μm. Statistical significance was determined using a one-way ANOVA with Tukey’s correction for multiple comparisons in **(B–E)** and an unpaired two-tailed *t* test in **(G, I)** Data are represented as mean ± standard error of mean (SEM). For **(B–D)**, significant changes compared to Monocytes: *p < 0.05, **p < 0.01, ***p < 0.001, and ****p < 0.0001; significant changes compared to Macrophages: ^###^*p* < 0.001, and ^####^*p* < 0.0001. For **(E)**, *p < 0.05, **p < 0.01, ***p < 0.001, and ****p < 0.0001.

PEGylated liposomes have been widely used for drug delivery, extending the half-life and controlling the release of encapsulated drugs (36, 37). They can deliver drugs to the desired target through passive and/or active targeting, thus avoiding off-target side effects and improving the therapeutic benefits (38). Many factors, such as size, charge, and composition, determine the efficacy and targeting of liposomes (39–41). Previous studies have shown that liposome size can shape immune responses, with larger particles preferentially targeting macrophages (42, 43). Therefore, we examined how PEGylated liposome size influences targeting of macrophages and other immune cells in the spinal cord during EAE. We fluorescently labeled 150, 550, and 700 nm PEGylated liposomes with fluorescein isothiocyanate (FITC) and began treatment two days after disease induction (**Figure 2A**). Although 150 nm PEGylated liposomes primarily targeted monocytes and macrophages, most of these cells were not FITC-positive (**Figure 2B**). In contrast, larger PEGylated liposomes (500 and 700 nm) more effectively and selectively targeted CNS-infiltrating monocytes and macrophages, increasing FITC positivity by approximately two-fold compared to 150 nm PEGylated liposomes (**Figures 2C, D**). Notably, the PEGylated liposomes failed to target CNS-resident microglia, suggesting that PEGylated liposomes primarily target monocytes in the periphery before their infiltration into the spinal cord during EAE.

We also tracked the disease severity of mice treated prophylactically with PEGylated liposomes of different sizes. Treatment with the larger (~700 nm) PEGylated liposomes effectively prevented EAE development, as treated mice exhibited no neurological manifestations (**Figure 2E**), minimal demyelination (**Figures 2F, G**), and reduced immune infiltration (**Figures 2H, I**) at day 15 post-induction compared to control mice. There were no differences in body weight (**Supplementary Figure S5A**) or the weights of the kidneys, heart, or brain (**Supplementary Figures S5B–I**) between control and liposome-treated mice. Liposome-treated mice displayed a slight, but not statistically significant, increase in liver weight (**Supplementary Figures S5J, K**), but the livers showed no signs of hepatotoxicity (**Supplementary Figure S5L**). Based on these results, we utilized the large macrophage-targeted PEGylated (L-PEGylated) liposomes in subsequent experiments.

### 3.4 Treatment with macrophage-targeted PEGylated liposomes after the onset of EAE attenuates disease progression and suppresses immune cell infiltration into the spinal cord

**Figure 3:**
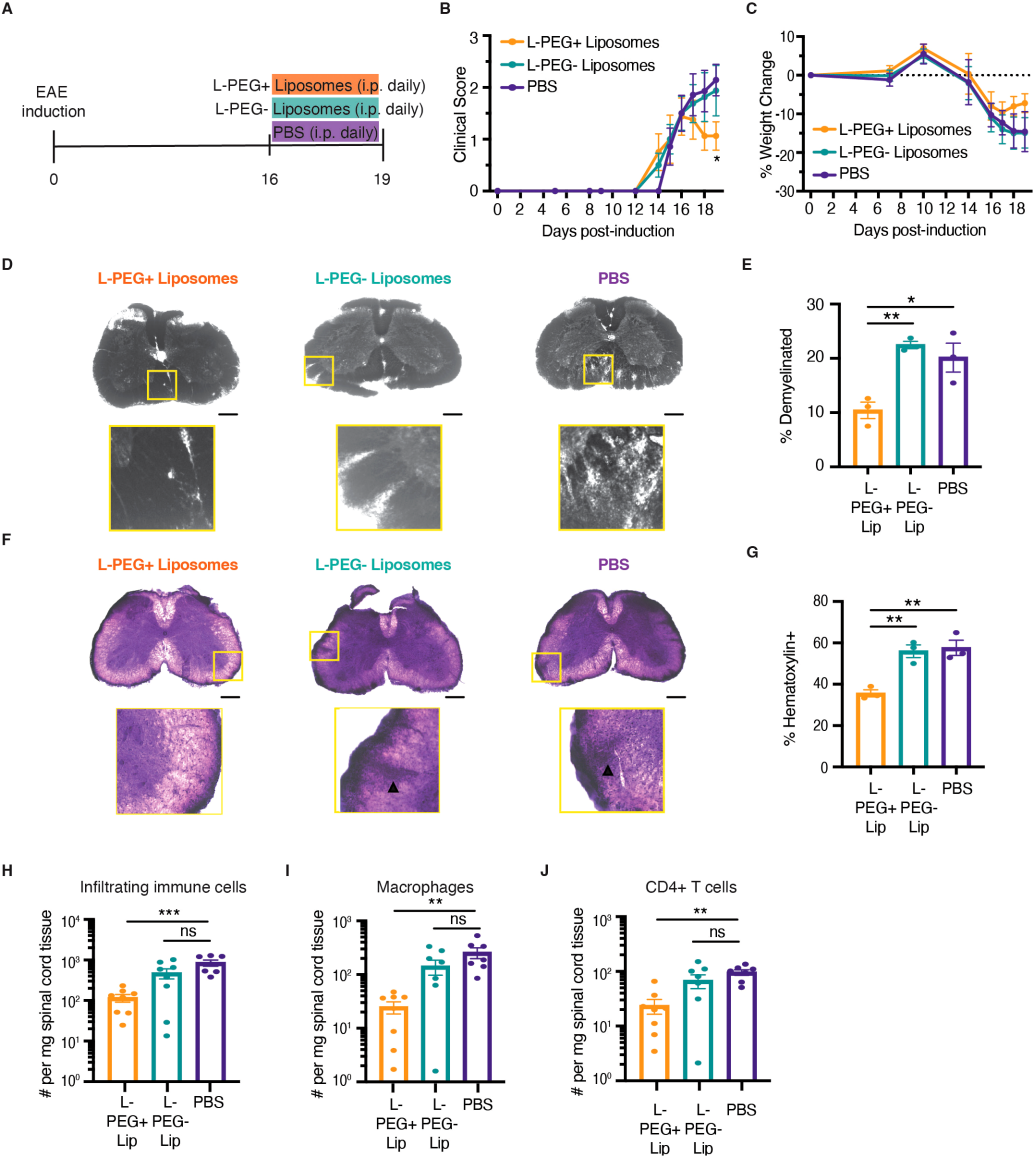
Treatment with macrophage-targeted PEGylated liposomes after the onset of disease attenuates EAE progression and suppresses immune cell infiltration into the spinal cord. **(A)** Schematic diagram of daily therapeutic L-PEGylated (L-PEG+) liposome treatment starting day 16 post-EAE induction, when mice had an average score of about 1.5. Control groups included mice treated with large nonPEGylated (L-PEG-) liposomes or PBS (untreated). Mice were monitored for neurological manifestations **(B)** and weight change **(C)** over the course of treatment (n=7–8 per group). **(D–G)** Spinal cords from L-PEG+ liposome-treated, L-PEG- liposome-treated, or untreated mice were isolated on day 19 post-EAE induction. **(D, E)** Demyelination was assessed using Sudan Black B (SBB) staining and **(F, G)** immune cell infiltration was assessed using H&E staining within the white matter on fixed frozen sections (n=3 per group). Triangles denote regions of dense immune cell infiltration. Scale bar, 200 μm. The absolute numbers of infiltrating immune cells **(H)**, macrophages **(I)**, and CD4+ T cells **(J)** in the spinal cords on day 19 post-EAE induction were assessed using flow cytometry (n=7–8 per group). Statistical significance was determined using a two-way ANOVA with Šidák multiple comparisons in **(B, C)**, an unpaired two-tailed *t* test in **(E, G)**, and a one-way ANOVA with Tukey’s correction for multiple comparisons in **(H**–**J)**. Data are represented as mean ± standard error of mean (SEM). Significant changes compared to PBS: *p < 0.05, **p < 0.01, and ***p < 0.001.

When given prophylactically, the L-PEGylated liposomes were superior to PEG alone in preventing EAE development (**Figures 1** and **2**). Because MS patients only initiate treatment after diagnosis, when the disease is already established (44), we next tested whether L-PEGylated liposomes exhibited therapeutic potential when administered to EAE mice after disease onset, compared to similarly sized non-PEGylated liposomes or PBS (**Figure 3A**). Only mice treated with L-PEGylated liposomes exhibited a decrease in neurological manifestations (**Figure 3B**), a reversal of weight loss (**Figure 3C**), and reduced demyelination (**Figures 3D, E**) and immune infiltration (**Figures 3F, G**). We further evaluated their efficacy in the relapsing-remitting EAE model in SJL/J mice. Following EAE induction, mice were treated daily with L-PEGylated liposomes from days 13 to 25 post-induction. Mice treated with the L-PEGylated liposomes remained relapse-free throughout treatment and only started relapsing 8 days after cessation of treatment (**Supplementary Figure S6**).

The development and progression of EAE are driven by increased infiltration of immune cells, particularly monocytes/macrophages and CD4+ T cells, into the spinal cord (45, 46). To investigate the effect of L-PEGylated liposomes on immune cell infiltration, we isolated the spinal cords from mice treated with these liposomes following disease onset. Only mice treated with L-PEGylated liposomes exhibited a significant decrease in the absolute number of infiltrating immune cells (**Figure 3H**), including macrophages (**Figure 3I**; **Supplementary Figures S7A, B**). Despite preferentially targeting monocytes and macrophages (**Figure 2D**), L-PEGylated liposomes also significantly reduced the absolute number of CD4+ T cells (**Figure 3J**; **Supplementary Figures S7C, D**). These results demonstrate that macrophage-targeted PEGylated liposomes administered following the onset of active disease reduce immune cell infiltration and reverse disease manifestations.

### 3.5 IL-1β secretion by macrophages is dampened by macrophage-targeted PEGylated liposomes

**Figure 4:**
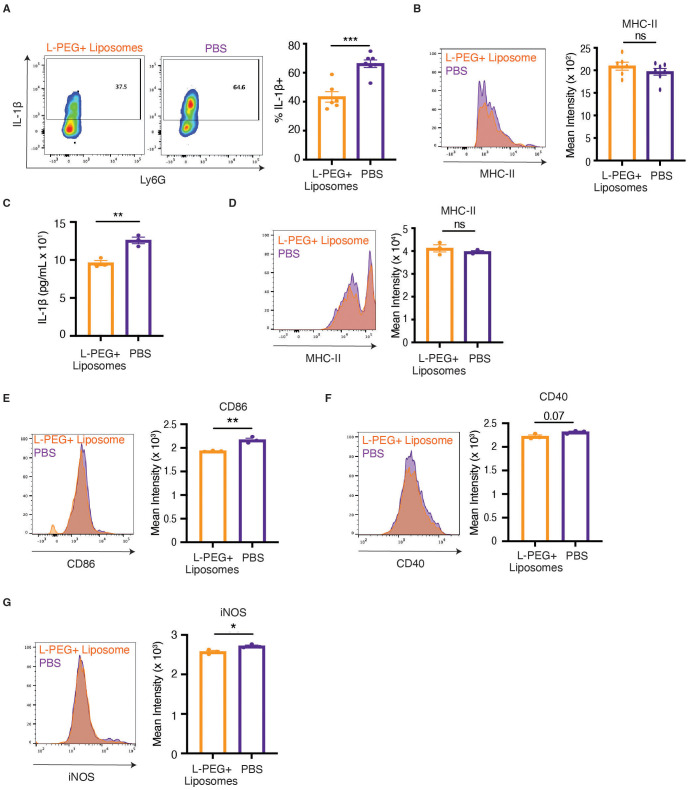
IL-1β secretion by macrophages is dampened by macrophage-targeted PEGylated liposomes. **(A, B)** After EAE induction, mice were treated daily with L-PEG+ liposomes or PBS starting day 16 post-EAE induction, and spinal cord cells were isolated on day 19 post-induction. Cells were stained for **(A)** intracellular IL-1β expression after a 4 hour stimulation with LPS or **(B)** MHC-II expression (n=6–7 per group). **(C)** BMDMs were pre-treated with L-PEG+ liposomes or PBS for 4 hours and then stimulated with LPS for 18 hours. The concentration of IL-1β in the supernatant was then assessed using flow cytometry-based cytokine bead array (n=3 technical replicates per group). **(D–G)** BMDMs were pre-treated with large PEGylated (L-PEG+) liposomes or untreated for 4 hours, stimulated with LPS for 18 hours, and then their expression of MHC-II **(D)**, CD86 **(E)**, CD40 **(F)**, and iNOS **(G)** were assessed using flow cytometry (n=3 technical replicates per group). Statistical significance was determined using an unpaired two-tailed *t* test in **(A–G)**. Data are represented as mean ± standard error of mean (SEM). *p < 0.05, **p < 0.01, and ***p < 0.001.

Macrophages are key orchestrators of the inflammatory response of other immune cells, like T cells, through their potent antigen presentation capabilities and cytokine secretion (9–11). Because pro-inflammatory cytokines such as IL-1β play central roles in promoting neuroinflammation and demyelination during EAE (47, 48), we first examined cytokine expression in spinal cord-infiltrating macrophages following treatment. Macrophages isolated from mice treated with L-PEGylated liposomes displayed a significant reduction in intracellular IL-1β following ex vivo stimulation (**Figure 4A**). In contrast, MHC-II expression on these cells remained unchanged (**Figure 4B**), suggesting that antigen presentation capacity was largely unaffected. These findings indicated that the therapeutic efficacy of L-PEGylated liposomes may lie in its modulation of cytokine production, rather than changes in antigen presentation, in macrophages.

To determine whether this reflected a direct effect on macrophages, we next assessed the impact of L-PEGylated liposomes on BMDMs *in vitro*. Pretreatment of BMDMs with L-PEGylated liposomes followed by overnight LPS stimulation significantly reduced IL-1β secretion (**Figure 4C**), whereas IL-6 and TNF-α production were unchanged (**Supplementary Figure S8**). We then evaluated whether these effects were accompanied by alterations in macrophage activation phenotype. The liposome-treated BMDMs expressed similar levels of MHC-II (**Figure 4D**) and exhibited modest decreases in canonical M1 markers, including CD86, CD40, and iNOS (**Figures 4E–G**), as well as M2 markers, including CD206 and Arg-1 (**Supplementary Figure S9**), compared to untreated BMDMs. Cell size, which can influence macrophage polarization (49), was also unchanged (**Supplementary Figure S10**). Together, these data demonstrate that macrophage-targeted PEGylated liposomes do not broadly reprogram macrophage activation but instead selectively suppress IL-1β production in macrophages, which may contribute to their therapeutic efficacy.

## 4 Discussion

PEG is widely used in the clinic to improve the pharmacokinetic and pharmacodynamic properties of therapeutic agents, enabling drugs like interferon β1a to be administered less frequently while maintaining efficacy in relapsing-remitting MS (50). Although PEG is often regarded as a biologically inert modifier, accumulating evidence indicates that it possesses intrinsic anti-inflammatory and membrane-stabilizing properties (27–30). These effects likely depend on direct interaction between PEG and cellular components, which may be limited when PEG is covalently linked to another molecule and could explain why PEGylated and nonPEGylated interferon β1a exhibit comparable efficacy (51). In neuroinflammatory models, PEG has been reported to reduce demyelination in EAE when administered early with polysorbate-80, which facilitates blood-brain barrier (BBB) transport (31). Our findings show that systemic administration of PEG alone did not significantly affect disease severity, suggesting that effective modulation of neuroinflammation may require delivery systems that enhance PEG transport into the CNS or target CNS-infiltrating immune effector cells.

Given that PEG attenuates pro-inflammatory cytokine secretion, we hypothesized that PEG might directly suppress macrophage activation. Previous studies reported that PEGylated nanoparticles reduced cytokine and chemokine expression in macrophages *in vitro* (35), but the specific contribution of PEG versus the other nanoparticle components remained unclear. In this study, we demonstrated for the first time that macrophage-targeting PEGylated liposomes significantly suppress EAE severity and reduce macrophage IL-1β expression more effectively than PEG alone.

The precise mechanism underlying PEG’s anti-inflammatory effects in macrophages remains to be fully elucidated. Although a previous study suggested that PEG can inhibit NF-κb signaling (35), we observed minimal changes in canonical M1 and M2 markers, accompanied by a selective reduction in IL-1β secretion, without alterations in other pro-inflammatory cytokines such as IL-6 or TNF-α. These findings indicate that PEG does not broadly suppress macrophage activation but instead primarily affects IL-1β secretion. PEG has been reported to modulate NLRP3 inflammasome activation (52, 53), which regulates IL-1β maturation and release. This effect may occur through PEG’s membrane-stabilizing properties that limit cellular stress and membrane disruption or through interactions with intracellular components upstream of inflammasome activation. Notably, global deletion of IL-1β or IL-1 receptor signaling markedly attenuates EAE (47), and multiple studies implicate myeloid-derived IL-1β as a key driver of neuroinflammation (54, 55). Given the central role of IL-1β in promoting neuroinflammation (48), its selective suppression by PEG may underlie the observed therapeutic effects.

IL-1β plays a central role in driving both demyelination and immune cell infiltration in MS (47, 48). Elevated IL-1β inhibits oligodendrocyte precursor cell (OPC) differentiation into mature myelin-producing oligodendrocytes (56). Beyond its direct effects on OPCs, IL-1β induces astrocyte reactivity, enhancing the release of inhibitory mediators that further impede OPC maturation (57, 58), and skews T cells towards the pathogenic Th17 subset that can directly damage oligodendrocytes (59, 60). Together, these effects lock OPCs in a precursor state and perpetuate failure of remyelination and tissue repair. In addition to its effects on myelination, IL-1β promotes leukocyte infiltration into the CNS by increasing BBB permeability through endothelial activation, chemokine release, and upregulation of adhesion molecules like ICAM-1 and VCAM-1 (61–64). By suppressing IL-1β secretion by macrophages, PEGylated liposomes may both restore conditions favorable for OPC differentiation and myelin repair and limit inflammation-driven CNS injury.

In the relapsing-remitting EAE model in SJL/J mice, treatment with PEGylated liposomes delayed, but did not fully prevent, disease relapse, indicating that the therapeutic effect may be transient. Even after treatment cessation, mice remained relapse-free for at least seven days, suggesting that dosing once or twice per week could sustain remission. This likely reflects the finite *in vivo* half-life of the liposomes, after which their anti-inflammatory effects wane. Importantly, treatment caused no overt systemic toxicity, with only a mild (>15%) increase in liver weight, consistent with normal hepatic uptake of liposomes (65). However, closer examination of the livers showed no significant histopathological toxicity. Given that less frequent dosing may maintain efficacy, this approach could further limit any potential liver-associated effects. Future studies should evaluate whether optimized dosing schedules or sustained-release formulations can more effectively prevent relapse while maintaining safety.

Taken together, our results demonstrate that macrophage-targeted PEGylated liposomes modulate both immune responses and disease manifestations in EAE. Coupled with the well-established clinical safety of PEGylated liposomes, these findings support their therapeutic potential in MS. Prior studies have shown efficient uptake of ligand-targeted liposomes by human macrophages (66, 67), as well as suppression of pro-inflammatory cytokine production by liposomes carrying immunomodulatory agents (68, 69), highlighting the translational feasibility of this approach. Importantly, our data indicate that PEGylated liposomes reduce IL-1β-mediated neuroinflammation even without targeting ligands or additional drugs, although incorporation of anti-inflammatory or tolerogenic agents could further enhance macrophage modulation and prolong therapeutic effects (70). Given IL-1β‘s role in other neurodegenerative diseases (48, 71, 72), including Alzheimer’s and Parkinson’s disease, macrophage-targeted PEGylated liposomes may represent a broader strategy for treating multiple neuroinflammatory and neurodegenerative disorders.

## Data Availability

The original contributions presented in the study are included in the article/**Supplementary Material**. Further inquiries can be directed to the corresponding authors.
