# Supplementary figures and images for: Macrophage-targeted PEGylated liposomes ameliorate experimental autoimmune encephalomyelitis
Source: Front Immunol. 2026 Jan 15;16:1657131. doi: 10.3389/fimmu.2025.1657131 (PMC12852013; doi:10.3389/fimmu.2025.1657131)

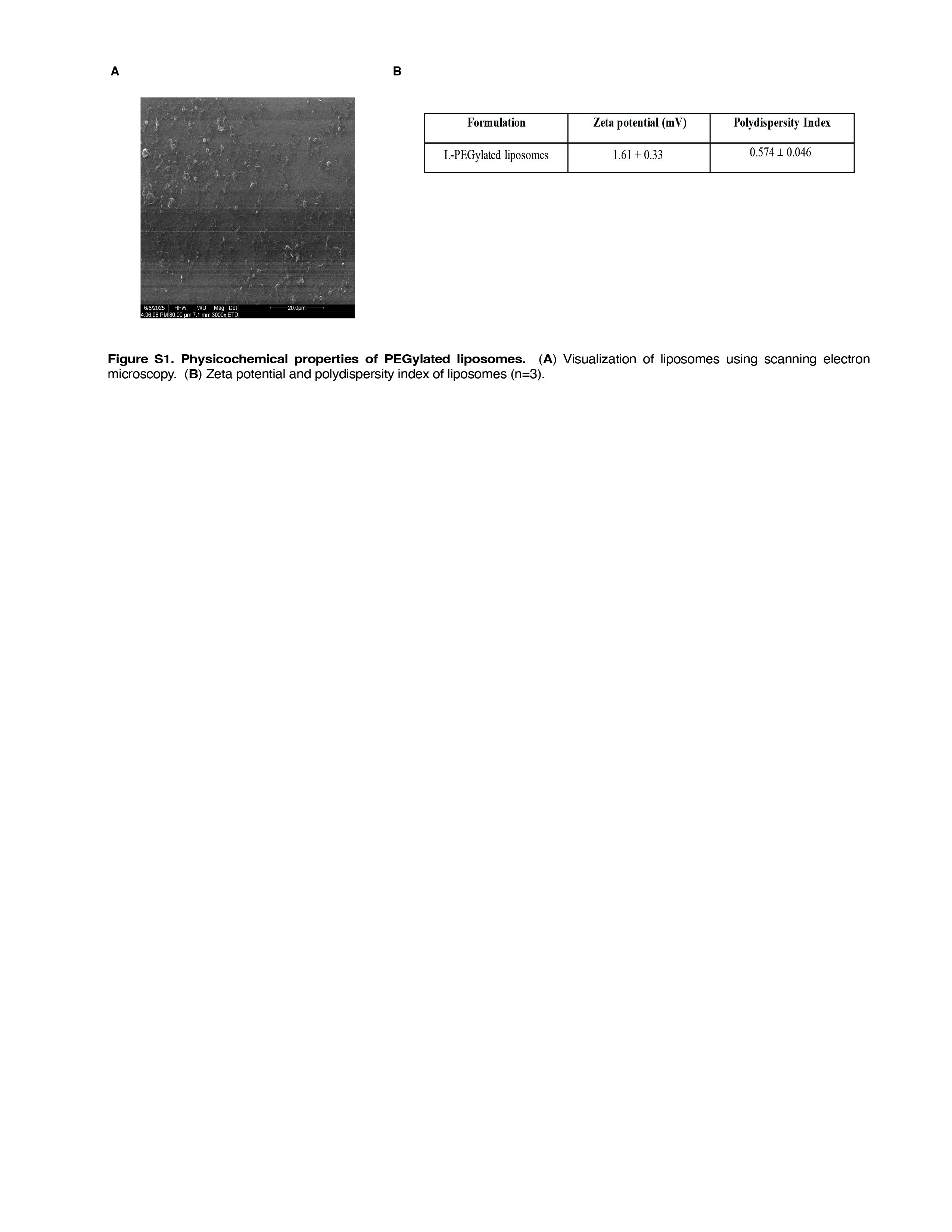

Supplement: Supplementary file 1 [file Image1.jpeg]

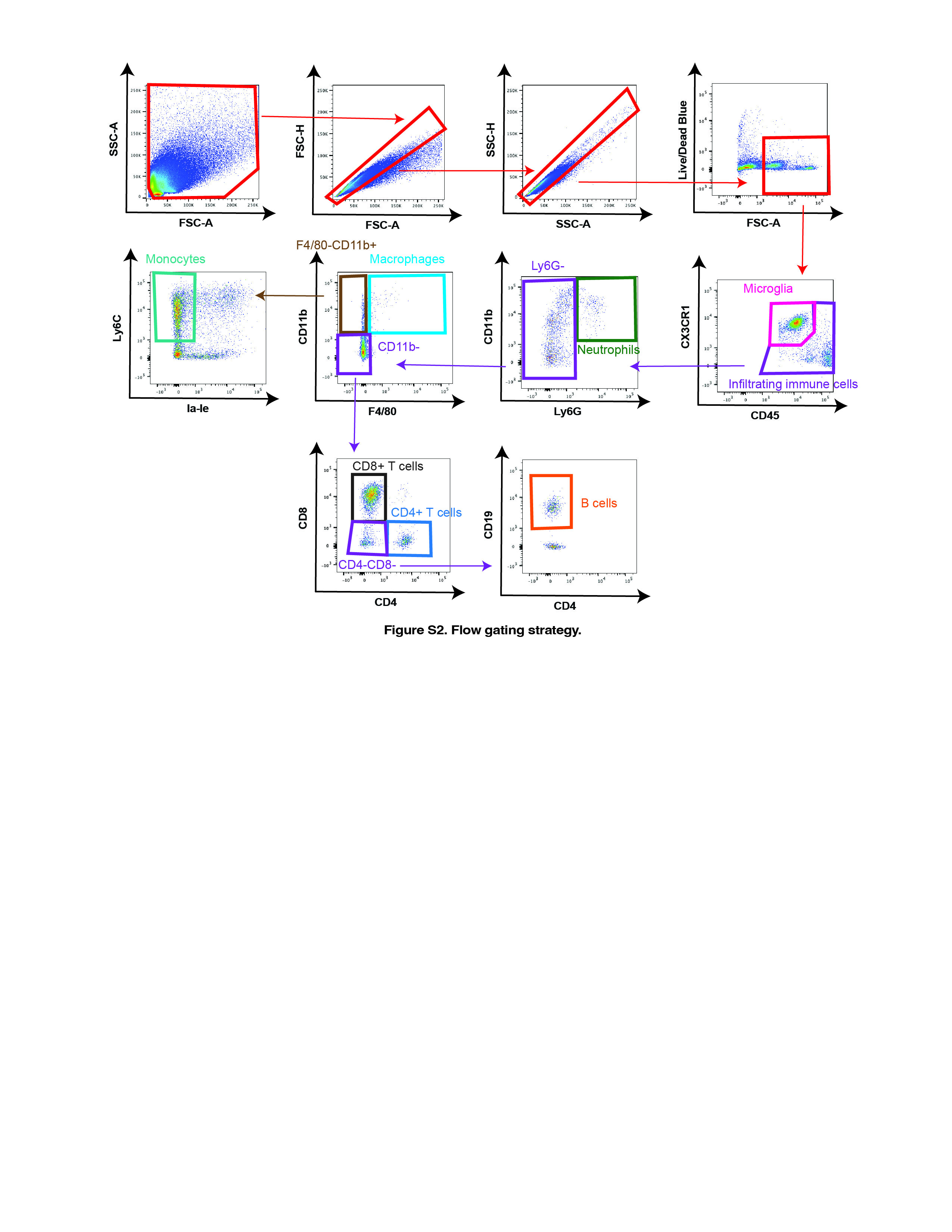

Supplement: Supplementary file 2 [file Image2.jpeg]

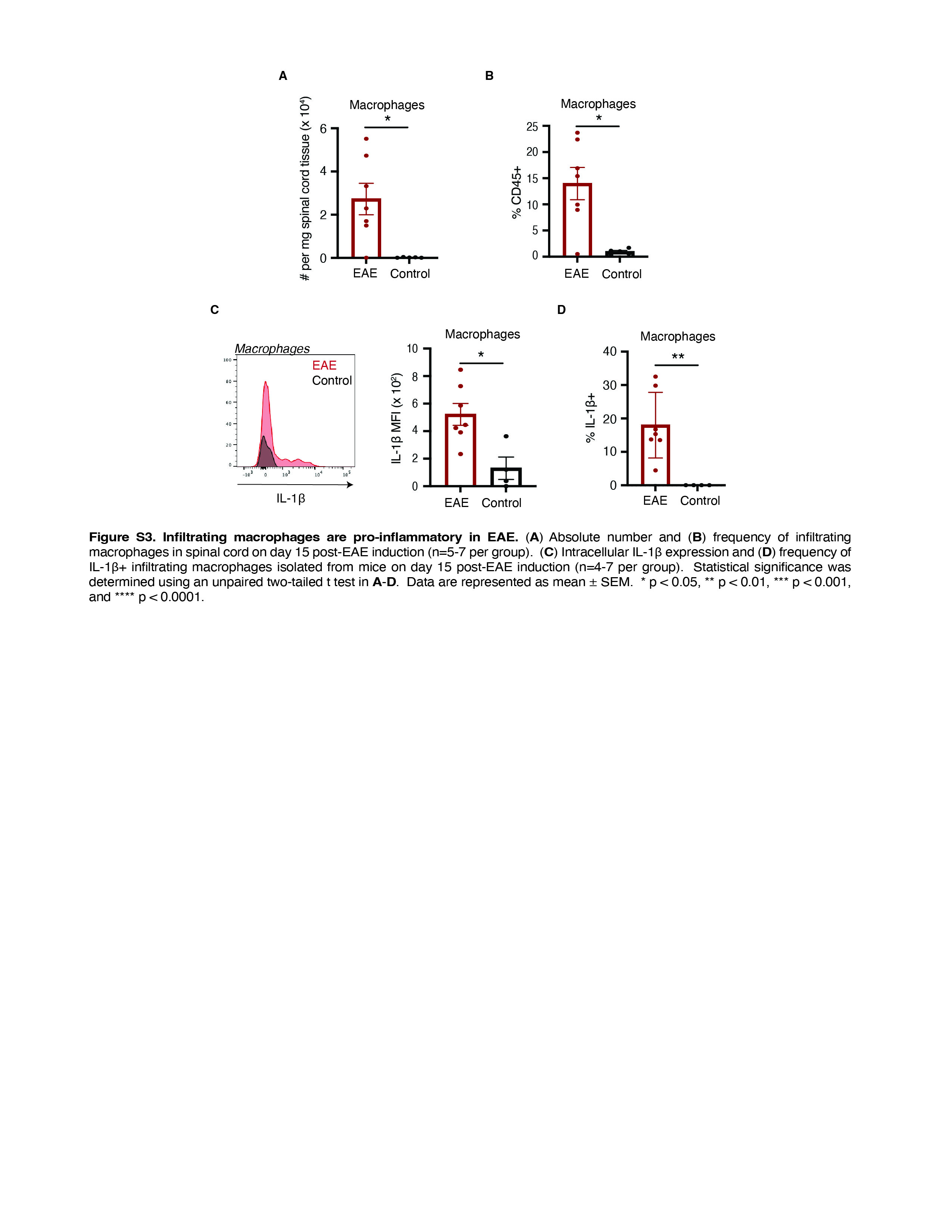

Supplement: Supplementary file 3 [file Image3.jpeg]

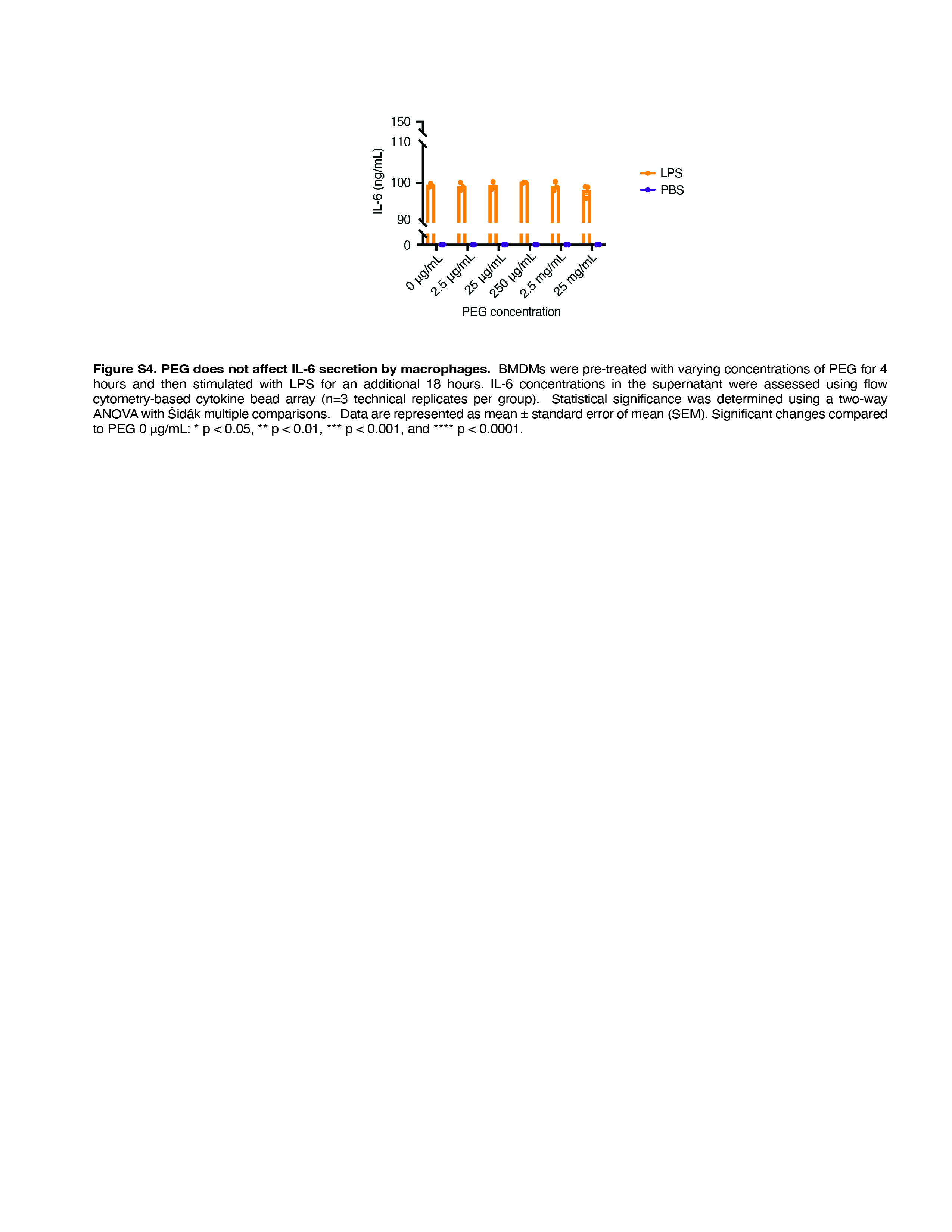

Supplement: Supplementary file 4 [file Image4.jpeg]

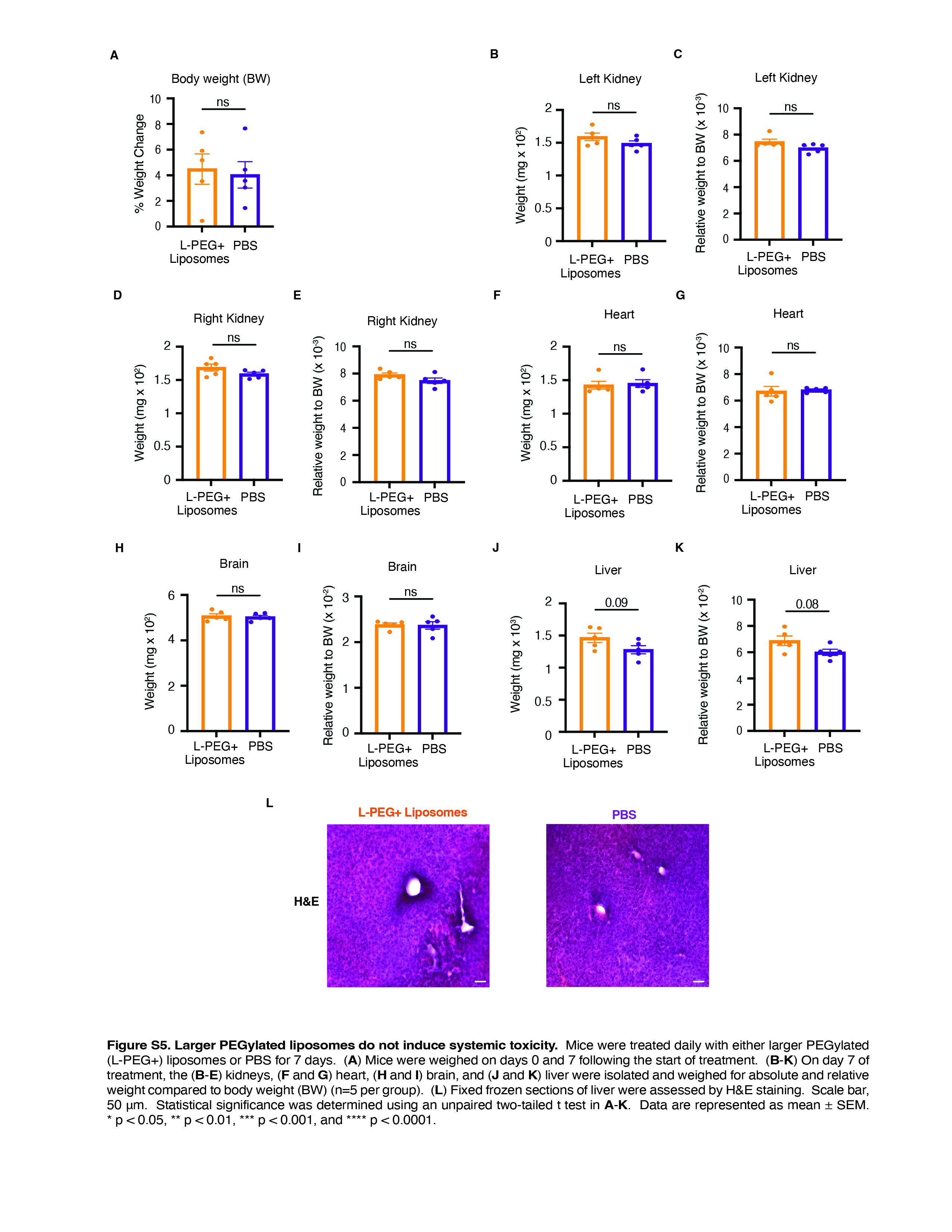

Supplement: Supplementary file 5 [file Image5.jpeg]

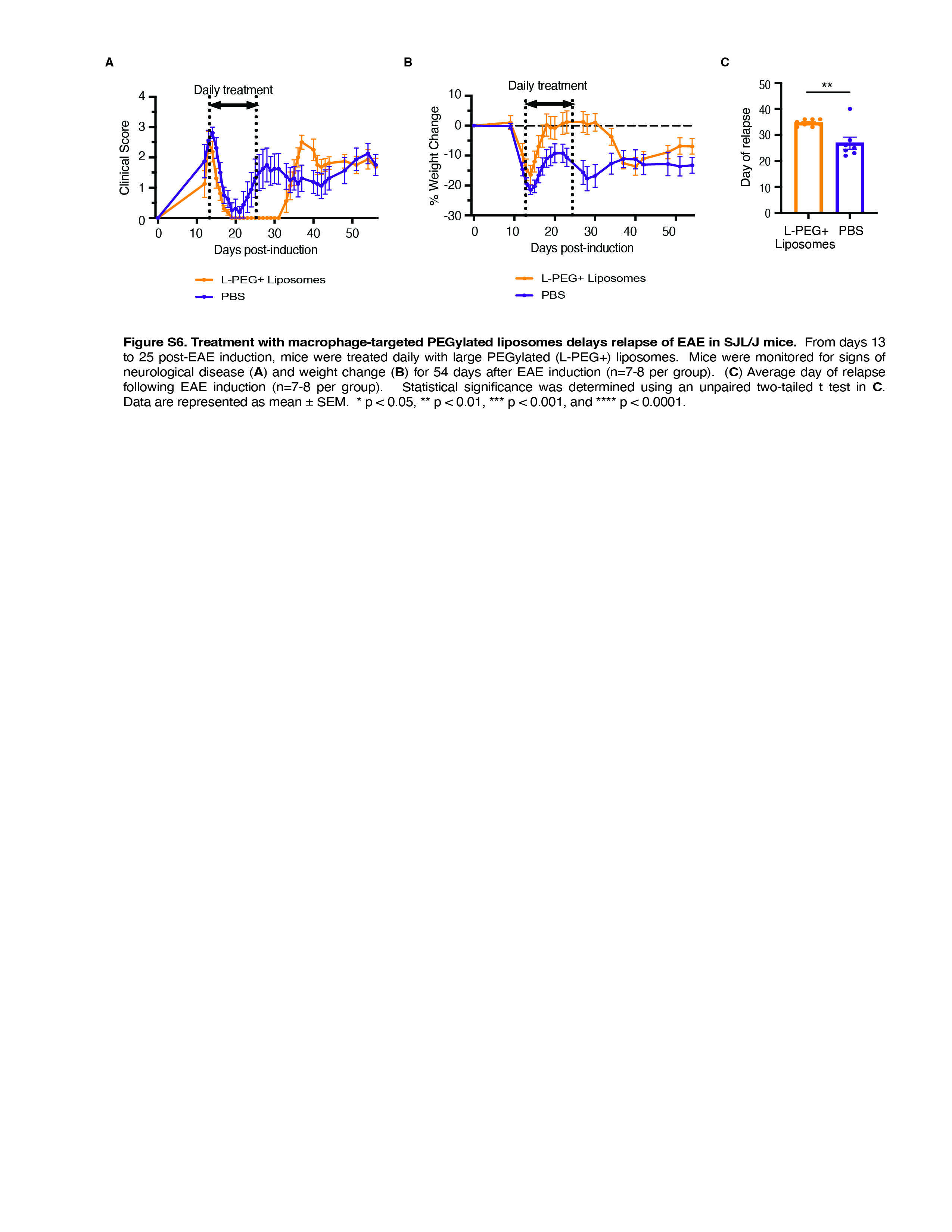

Supplement: Supplementary file 6 [file Image6.jpeg]

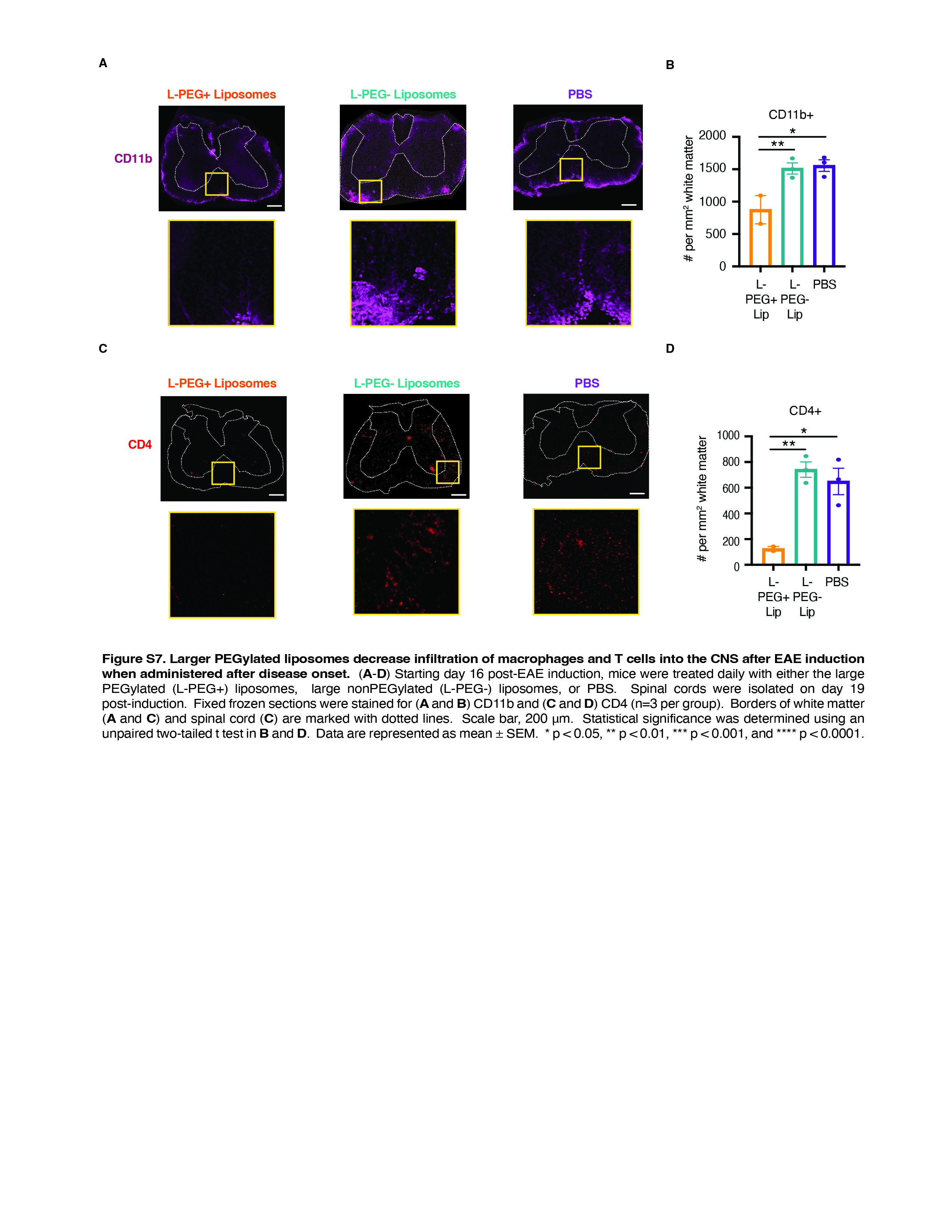

Supplement: Supplementary file 7 [file Image7.jpeg]

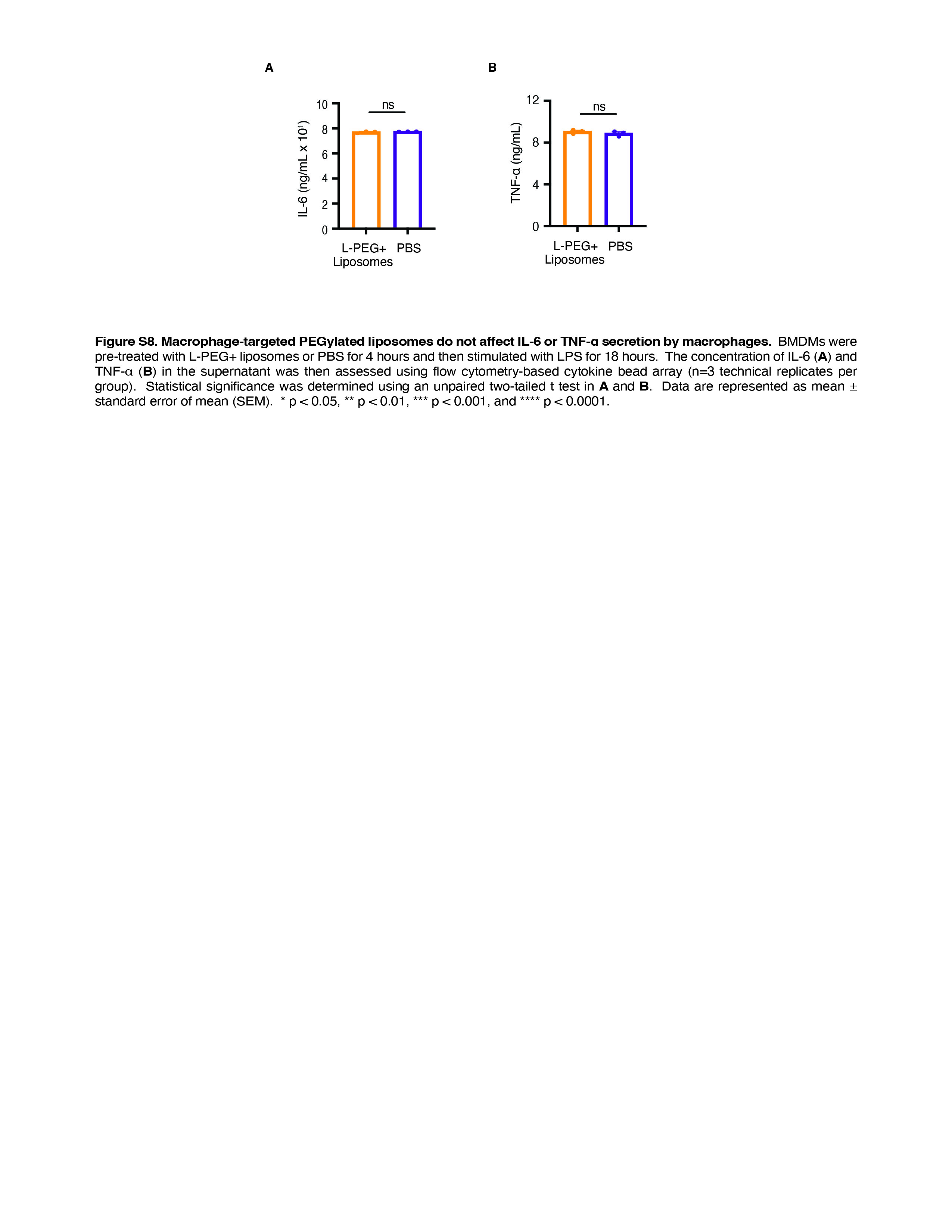

Supplement: Supplementary file 8 [file Image8.jpeg]

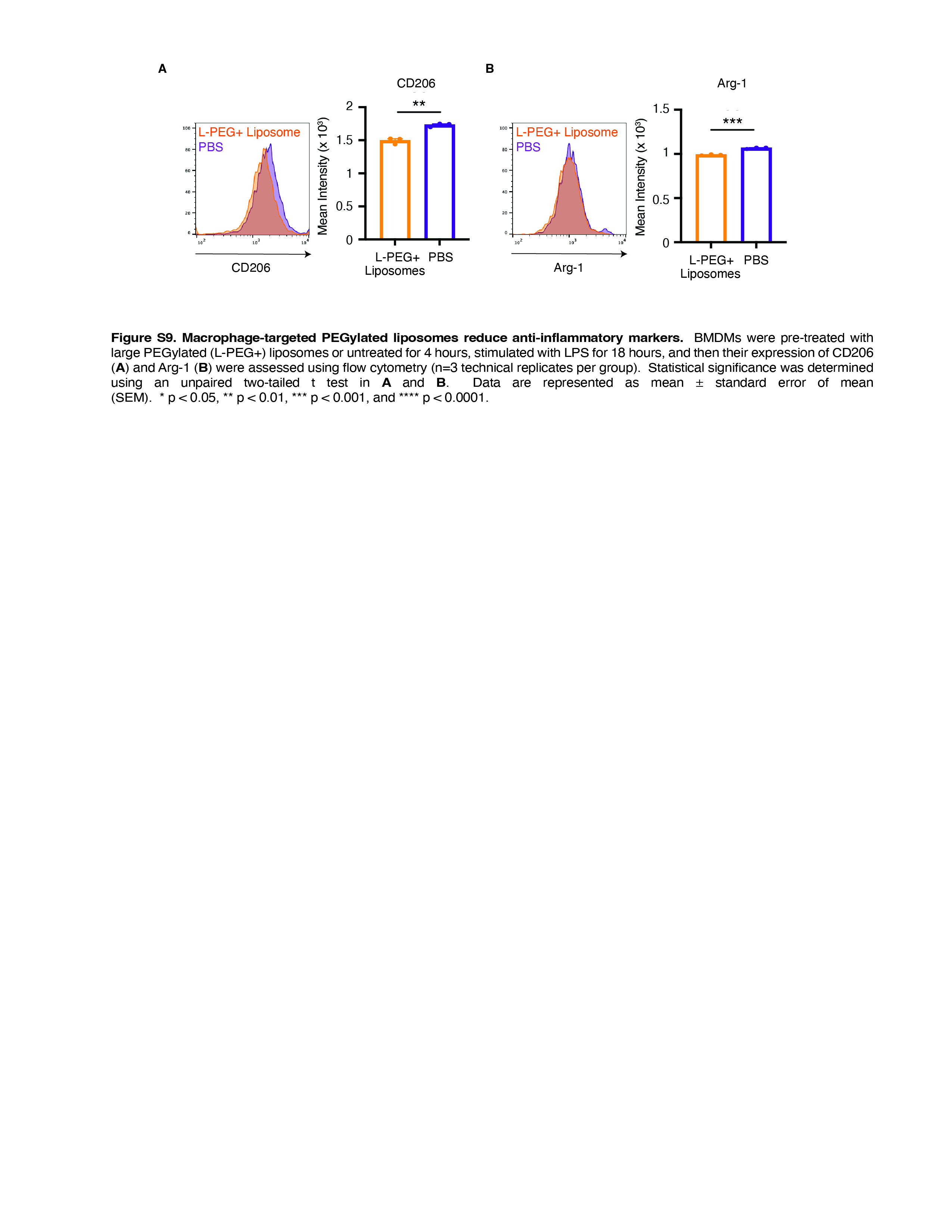

Supplement: Supplementary file 9 [file Image9.jpeg]

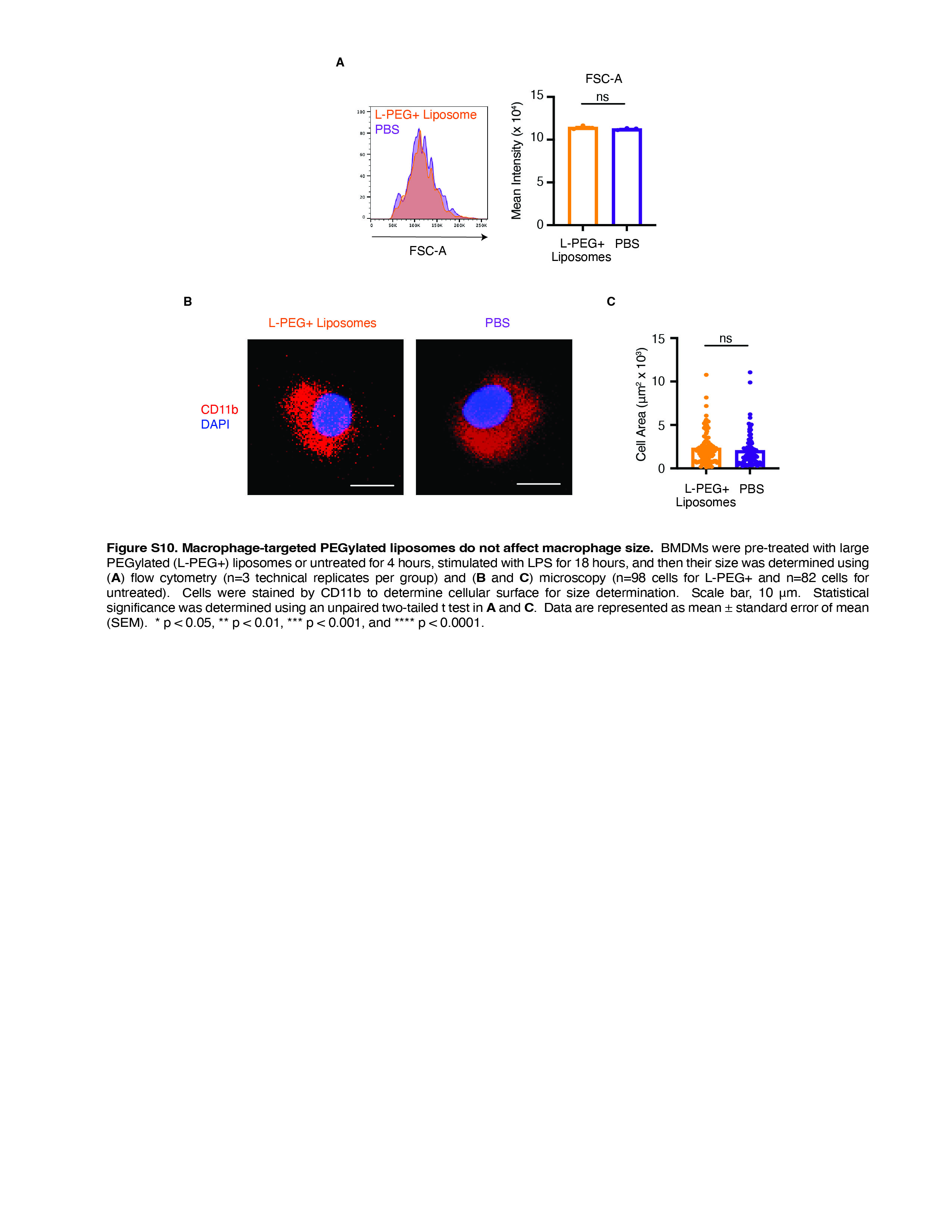

Supplement: Supplementary file 10 [file Image10.jpeg]

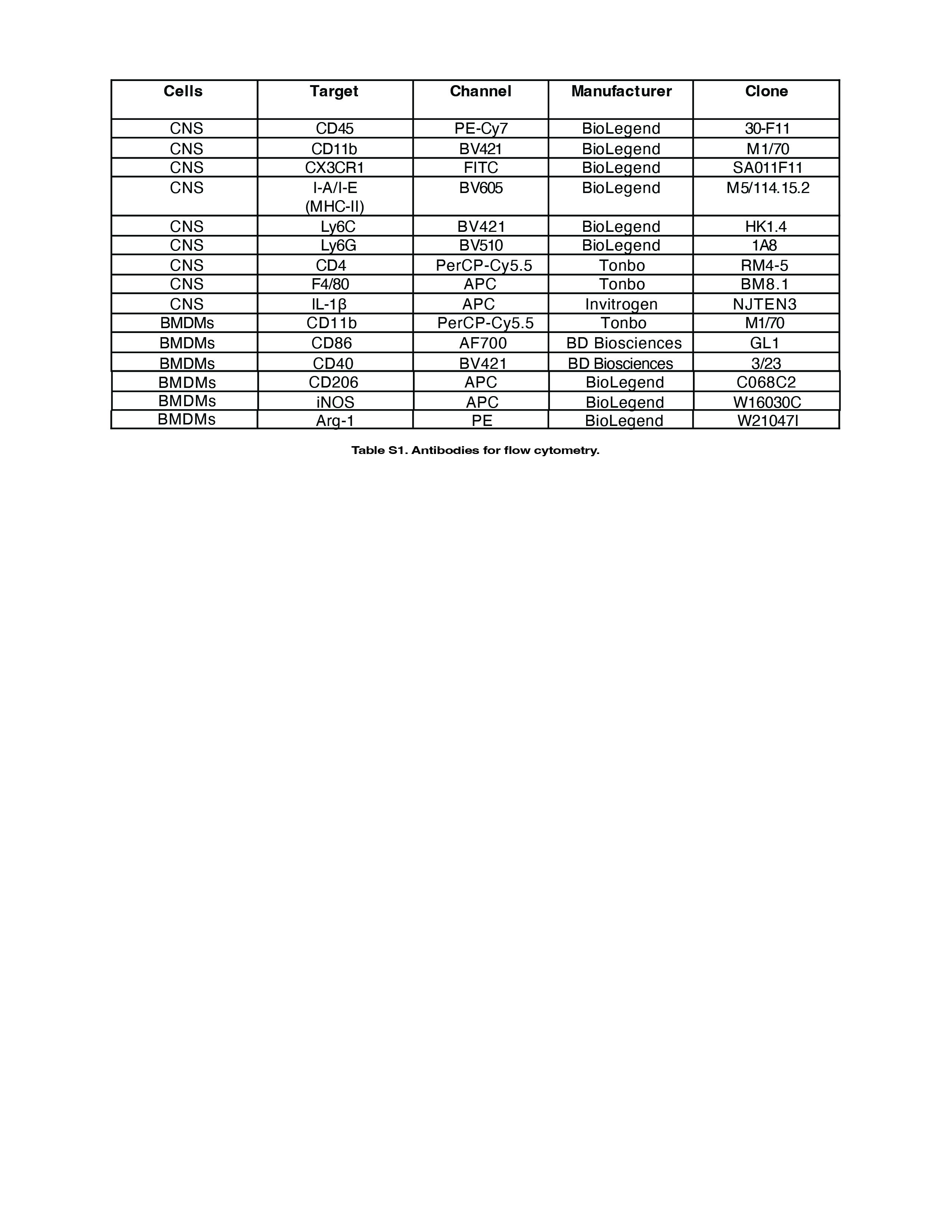

Supplement: Supplementary file 11 [file Image11.jpeg]
